# Bloodstream Infection due to Piperacillin/Tazobactam Non-Susceptible, Cephalosporin-Susceptible *Escherichia coli*: A Missed Opportunity for De-Escalation of Therapy

**DOI:** 10.3390/antibiotics7040104

**Published:** 2018-12-01

**Authors:** Leah Carlisle, Julie Ann Justo, Majdi N. Al-Hasan

**Affiliations:** 1 College of Pharmacy, University of South Carolina, Columbia, SC 29208, USA; leahc@email.sc.edu; 2 Department of Clinical Pharmacy and Outcomes Sciences, College of Pharmacy, University of South Carolina, Columbia, SC 29208, USA; justoj@cop.sc.edu; 3 Department of Pharmacy, Palmetto Health Richland, Columbia, SC 29203, USA; 4 School of Medicine, University of South Carolina, Columbia, SC 29209, USA; 5 Department of Medicine, Division of Infectious Diseases, Palmetto Health University of South Carolina Medical Group, Columbia, SC 29203, USA

**Keywords:** Bacteremia, sepsis, pyelonephritis, antibiotics, antimicrobial stewardship, beta-lactam/beta-lactamase inhibitors, ESBL, TEM-1

## Abstract

An increasing number of reports describing *Escherichia coli* isolates with piperacillin/tazobactam resistance, despite retained cephalosporin susceptibility, suggest further emergence of this phenotypic resistance pattern. In this report, a patient with metastatic breast cancer presented to medical care after two days of chills, nausea, vomiting, reduced oral intake, and generalized weakness. Blood and urine cultures grew *E. coli* as identified by rapid diagnostics multiplex PCR and MALDI-TOF, respectively. The patient continued to manifest signs of sepsis with hypotension and tachypnea during the first three days of hospitalization despite empirical antimicrobial therapy with intravenous piperacillin/tazobactam. After in vitro antimicrobial susceptibility testing demonstrated a piperacillin/tazobactam minimal inhibitory concentration (MIC) of 64 and a ceftriaxone MIC of ≤1 mcg/mL, antimicrobial therapy was switched from intravenous piperacillin/tazobactam to ceftriaxone. All symptoms and signs of infection resolved within 48 h of starting ceftriaxone therapy. This report describes the clinical failure of piperacillin/tazobactam in the treatment of a bloodstream infection due to *E. coli* harboring a phenotypic resistance pattern of isolated piperacillin/tazobactam non-susceptibility. The case demonstrates the role of cephalosporins as potential treatment options and highlights the value of early de-escalation of antimicrobial therapy based on rapid diagnostic testing for microbial identification.

## 1. Case

A woman over 70 years old presented to a local emergency room in the summer of 2017 with a 2-day history of chills, nausea, vomiting, reduced oral intake, and generalized weakness. She was unable to get out of bed over the past 24 h. Upon review of systems, she had no urinary symptoms, abdominal pain, diarrhea, or other complaints. Past medical history was remarkable for metastatic breast cancer, hypertension, and depression. Her home medications included oral letrozole, sertraline, amlodipine, and carvedilol. The patient was briefly hospitalized three months prior for new brain metastases. However, she did not receive antimicrobials or chemotherapy during that hospitalization or elsewhere over the past 6 months. She had no other known healthcare exposures or invasive procedures over the same period. The patient had no documentation of prior infections or colonization with multidrug-resistant bacteria. 

The patient had signs of sepsis on initial presentation with hypotension (blood pressure: 61/39 mmHg) and tachypnea (respiratory rate: 27 breaths/minute). Her tympanic temperature was 99.2 degrees Fahrenheit, and her heart rate was 76 beats per minute. She was alert and awake during the physical examination with dry skin and mucus membranes. A tunneled central venous port in the anterior chest had no erythema, drainage, or tenderness to touch. Significant laboratory findings included a peripheral white blood cell (WBC) count of 12,400/mm^3^ with bandemia (21%), elevated serum creatinine from baseline (2.9 mg/dL), and high serum procalcitonin (187 ng/mL). Urinalysis with microscopy demonstrated large leukocyte esterase, 95 WBCs with few WBC clumps, and 6 red blood cells. Rare bacteria were observed by urine microscopic examination. No new pulmonary infiltrates were noted on a chest X-ray.

The patient was admitted to the intensive care unit for management of suspected sepsis. Intravenous fluid resuscitation and empirical broad-spectrum antimicrobials (intravenous vancomycin and piperacillin/tazobactam) were started following collection of blood and urine cultures. An antimicrobial stewardship alert was triggered by the growth of Gram-negative bacilli on blood cultures after 12 h of collection. A BioFire FilmArray^®^ multiplex PCR Blood Culture Identification (BCID) panel detected *Enterobacteriaceae* and *Escherichia coli*. The *Klebsiella pneumoniae* carbapenemase (KPC) gene was not detected. A urine culture also grew *Escherichia coli* as identified by MALDI-TOF. At this point, the local antimicrobial stewardship team recommended discontinuation of vancomycin. Recommendations also included de-escalation of antimicrobial therapy from piperacillin/tazobactam to ceftriaxone given growth of *E. coli* in the bloodstream and the patient’s low risk of extended-spectrum beta-lactamases (ESBLs) given the absence of recent antimicrobial use or prior infections or colonization with ESBL-producing bacteria [1,2]. The primary team discontinued vancomycin; however, they preferred to continue intravenous piperacillin/tazobactam at 2.25 g every 6 h (dose adjusted for calculated creatinine clearance of 15 mL/min) until the availability of in vitro antimicrobial susceptibility testing results of the *E. coli* isolate. 

Following an initial relative improvement in hemodynamics with fluid resuscitation, the patient’s clinical response plateaued during the first three days of antimicrobial therapy. She continued to manifest signs of sepsis with a systolic blood pressure of <100 mmHg and a respiratory rate of >22 breaths/minute. On the fourth day of hospitalization, she developed a fever (102 degrees Fahrenheit) and new-onset diarrhea (5 watery bowel movements within 24 h), and her respiratory rate increased from 24–26 to 30 breaths/minute. *Clostridioides difficile* PCR was positive in a stool sample. Retroperitoneal ultrasound did not demonstrate ureteric obstruction or hydro-nephrosis. The in vitro antimicrobial susceptibility results of the *E. coli* bloodstream and urinary isolate are demonstrated in Table 1. Non-susceptibility to piperacillin/tazobactam via the automated VITEK^®^ 2 system was confirmed using the Kirby–Bauer method. In addition, ESBL screening by disk diffusion using cefotaxime/clavulanate combination disks was negative. The primary team requested a consultation with a specialist in Infectious Diseases for antimicrobial management.

## 2. Treatment Strategy and Patient Outcome

The Infectious Diseases consultation team initiated oral vancomycin for the treatment of the *C. difficile* infection. Piperacillin/tazobactam was discontinued and intravenous ceftriaxone at 2 g every 24 h was started for the treatment of the *E. coli* bloodstream infection secondary to a urinary source. The patient showed significant clinical improvement within 48 h of the new antimicrobial regimen. The fever, tachypnea, and hypotension all resolved. Antihypertensive medications were restarted due to consistent systolic blood pressure readings greater than 130 mmHg. The patient was discharged from the hospital after successful treatment of both infections with no evidence of recurrence in the subsequent 12 months. 

## 3. Discussion

*E. coli* is the most common bacterial cause of bloodstream infections (BSI) in North America and Europe [3]. A higher incidence rate in women than in men is due to predominance of the urinary tract as the source of *E. coli* BSI [4]. Appropriate empirical antimicrobial therapy is associated with improved survival in critically ill patients with BSI and a shorter hospital length of stay overall [5,6]. Increasing rates of antimicrobial resistance among *E. coli* bloodstream isolates have complicated empirical antimicrobial management [4,7]. Utilization of broad-spectrum antimicrobial agents, particularly piperacillin–tazobactam, has increased over the past two decades in United States hospitals [8]. The empirical use of piperacillin/tazobactam in patients with suspected Gram-negative BSI provides antimicrobial coverage for *Pseudomonas aeruginosa* and possibly ESBL-producing *Enterobacteriaceae* [9]. However, this “one-size-fits-all approach” may not be necessary beyond the first 24–48 h of presentation in the era of rapid diagnostics and prediction of antimicrobial resistance [1,2]. A recent study demonstrated that de-escalation of antimicrobial therapy to ceftriaxone is safe and effective once the bloodstream isolate has been identified as *E. coli*, in the absence of risk factors for ESBLs [1]. 

In the absence of molecular or PCR testing to determine the exact resistance mechanism, interpretation of the phenotypic resistance pattern is crucial to optimizing antimicrobial therapy. The *E. coli* isolate in this case is resistant to ampicillin, suggesting the presence of TEM-1 beta-lactamase. Non-susceptibility to both beta-lactam/beta-lactamase inhibitor combinations—amoxicillin/clavulanate and piperacillin/tazobatam—suggests either TEM-1 hyperproduction or an inhibitor resistance mechanism [10]. This is supported by the low minimal inhibitory concentrations (MICs) of the high-generation cephalosporins cefoxitin and ceftriaxone, which argue against the possibilities of AmpC beta-lactamases and ESBLs, respectively [11]. In addition, a negative ESBL screening test confirms the lack of ESBL production in this isolate.

This case demonstrates many benefits of early de-escalation of antimicrobial therapy in patients with *E. coli* BSI. First, in the era of increasing antimicrobial use and resistance, there are no guarantees that the broader-spectrum agent, piperacillin/tazobactam, includes antimicrobial coverage for more *E. coli* isolates than a narrower-spectrum agent, ceftriaxone. There are increasing numbers of reports describing the emergence of piperacillin/tazobactam resistance among *E. coli* isolates despite retained susceptibility to cephalosporins in over 20 states [12]. Tandem amplification of a complete transposon containing the bla-TEM-1 gene under selective antimicrobial pressure has been described as a potential explanation of this resistance pattern [13]. A recent study demonstrates relatively fair agreement between various automated in vitro antimicrobial susceptibility testing methods in identifying this phenotypic resistance [14]. It is speculated that increasing use of piperacillin/tazobactam in hospitals may have contributed to the emergence of isolated piperacillin/tazobactam resistance among *E. coli* isolates. This phenotypic resistance pattern does not appear to be uncommon. At our healthcare system of four community hospitals in central South Carolina, 3.1% (86/2733) of all-source *E. coli* isolates in the 2016 and 2017 calendar years were non-susceptible to piperacillin/tazobactam, despite retained susceptibility to ceftriaxone. Whereas piperacillin/tazobactam may add in vitro activity against *E. coli* isolates that harbor CTX-M-15 or other ESBLs, it may miss as many isolates with isolated piperacillin/tazobactam resistance, particularly in settings with a relatively low prevalence of ESBLs [2,9]. Second, early de-escalation of antipseudomonal beta-lactams, including piperacillin/tazobactam, in patients with *Enterobacteriacieae* BSI has been associated with a threefold reduction in the risk of *C. difficile* infection in a recent investigation [15].

Reports on optimal antimicrobial therapy for BSI due to *E. coli* with isolated piperacillin/tazobactam resistance remain scarce. A prior study, an animal model for treatment of such *E. coli* isolates, suggested that piperacillin/tazobactam may still be effective in vivo despite in vitro non-susceptibility to this agent. To our knowledge, the current report is the first to describe the clinical failure of piperacillin/tazobactam in the treatment of BSI due to piperacillin/tazobactam non-susceptible, cephalosporin-susceptible *E. coli*. The main limitation of this report is the lack of additional testing to determine the *E. coli* group or serotype, or to confirm the exact resistance mechanism. Further clinical studies on risk factors and optimal antimicrobial treatment options for infections due to *E. coli* isolates with this emerging phenotypic resistance pattern of isolated piperacillin/tazobactam resistance are warranted. 

## 4. Conclusions

*E. coli* resistance to piperacillin/tazobactam with retained susceptibility to cephalosporins is an emerging phenotypic resistance pattern. This case study describes clinical failure of piperacillin/tazobactam in the treatment of BSI and sepsis due to an *E. coli* with isolated piperacillin/tazobactam resistance. Rapid clinical response after switching therapy to ceftriaxone supports the use of in vitro antimicrobial susceptibility testing results in the selection of antimicrobials for treatment of BSI due to these *E. coli* isolates. The emergence of this phenotypic resistance pattern discourages the non-stratified utilization of broad-spectrum agents such as piperacillin/tazobactam for the empirical therapy of *E. coli* BSI. The report supports early de-escalation of antimicrobial therapy to ceftriaxone based on identification of *E. coli* in the bloodstream using rapid diagnostics in the absence of risk factors for ESBLs. Early de-escalation of antipseudomonal beta-lactams may also reduce the risk of *C. difficile* infection in patients with *Enterobacteriacieae* BSI.

## Figures and Tables

**Table 1:** In vitro antimicrobial susceptibility results of *E. coli* isolate.

Antimicrobial Agent	MIC	Categorical Interpretation
Ampicillin	≥32	Resistant
Amoxicillin/clavulanate	16	Intermediate
Piperacillin/tazobactam	64	Intermediate
Cefazolin	8	Variable interpretation *
Cefoxitin	≤4	Susceptible
Ceftriaxone	≤1	Susceptible
Cefepime	≤1	Susceptible
Ertapenem	≤0.5	Susceptible
Meropenem	≤0.25	Susceptible
Ciprofloxacin	≤0.25	Susceptible
Gentamicin	≤1	Susceptible
Trimethoprim/sulfamethoxazole	≥320	Resistant

MIC: Minimal inhibitory concentration in mcg/mL. * Despite similar cefazolin MICs, interpretation was resistant for the bloodstream isolate but susceptible for the urinary isolate due to different susceptibility breakpoints per the 2017 guidelines from the Clinical and Laboratory Standards Institute.
